# Experimental and Simulation Study on Failure of Thermoplastic Carbon Fiber Composite Laminates under Low-Velocity Impact

**DOI:** 10.3390/polym16182581

**Published:** 2024-09-12

**Authors:** Lei Yang, Xiaolin Huang, Zhenhao Liao, Zongyou Wei, Jianchao Zou

**Affiliations:** 1 College of Civil and Transportation Engineering, Shenzhen University, Shenzhen 518060, China; huangxiaolin2022@email.szu.edu.cn (X.H.); liaozhenhao2020@email.szu.edu.cn (Z.L.); weizongyou2022@email.szu.edu.cn (Z.W.); 2 Department of Mechanical and Automation Engineering, The Chinese University of Hong Kong, Hong Kong SAR 999077, China; jianchaozou@cuhk.edu.hk

**Keywords:** composites, low-velocity impact, progressive damage model, damage evolution

## Abstract

Numerous studies have demonstrated that under low-velocity, low-energy impact conditions, although the surface damage to fiber-reinforced composite laminates may be minimal, significant internal damage can occur. Consequently, a progressive damage finite element model was specifically developed for thermoplastic carbon fiber-reinforced composite laminates subjected to low-speed impact loads, with the objective of analyzing the damage behavior of laminates under impacts of varying energy levels. The model utilizes a three-dimensional Hashin criterion for predicting intralayer damage initiation, with cohesive elements based on bilinear traction–separation law for predicting interlaminar delamination initiation, and incorporates a damage constitutive model based on equivalent displacement to characterize fiber damage evolution, along with the B-K criterion for interlaminar damage evolution. The impact response of laminates at energy levels of 5 J, 10 J, 15 J, 20 J, and 25 J was analyzed through numerical simulation, drop-hammer experiments, and XCT non-destructive testing. The results indicated that the simulation outcomes closely correspond with the experimental findings, with both the predicted peak error and absorbed energy error maintained within a 5% margin, and the trends of the mechanical response curves aligning closely with the experimental data. The damage patterns predicted by the numerical simulations were consistent with the results obtained from XCT scans. The study additionally revealed that the impact damage of the laminates primarily stems from interlaminar delamination and intralayer tensile failure. Initial damage typically presents as internal delamination; hence, enhancing interlaminar bonding performance can significantly augment the overall load-bearing capacity of the laminate.

## 1. Introduction

Due to their exceptional specific strength and stiffness, high-temperature endurance, corrosion resistance, and design versatility, composite laminates are employed across various sectors, including aerospace, maritime, and automotive industries [1,2]. Nonetheless, during low-speed impacts, although the surface damage to laminated panels may appear inconspicuous, internal fractures and delamination have occurred, substantially reducing the panels’ performance and compromising the material’s safety [3]. Consequently, numerous researchers have made dedicated efforts to address the challenges posed by low-speed impacts on laminated panels.

In recent years, significant progress has been made in understanding the damage mechanisms of composites under a low-velocity impact, revealing how impact loads gradually weaken structural integrity through a series of progressive damage modes. These damage modes include transverse cracking, shear cracking, delamination, interfacial debonding, and, eventually, fiber fracture. During the impact process, the initial damage is usually transverse cracking, which occurs in planes perpendicular to the fiber direction. When the impact load exceeds the transverse strength limit of the material, these cracks form in the matrix or at the fiber/matrix interface [4]. As the stress increases, shear cracks occur along the direction of shear stress, typically forming in the interlaminar matrix of composites [5]. If the impact continues to increase, the damage further evolves into delamination, which is one of the most common failure modes under impact loads in composites, referring to the separation at the interlaminar interfaces of the material [6]. In more severe cases, interfacial debonding occurs, which refers to the failure of bonding between the fiber and the matrix. This typically happens during impact when the interfacial stress exceeds the bonding strength, leading to separation between the fiber and the matrix [7]. To better understand and predict these damages, researchers widely use non-destructive testing (NDT) techniques to detect and analyze internal damage in composites. For example, ultrasonic testing can effectively identify cracks and delamination in composites, while X-ray computed tomography (CT) provides three-dimensional images of structural damage. These techniques play an important role in damage assessment after low-velocity impacts. Wang et al.’s study [8] employed phased-array ultrasonic testing to comprehensively analyze the delamination mechanisms in thin composite plates subjected to low-velocity impact. They found that, particularly in fiber-reinforced polymers (FRPs), out-of-plane stresses and transverse shear often lead to delamination. Qiang et al. [9] used industrial CT scanning to investigate the internal crack propagation and delamination in carbon fiber/epoxy composites. They discovered that as the impact energy increases, significant long cracks, delamination, and “bridging” effects appear on the material’s surface and backside, with 45° shear fractures often occurring through the thickness, ultimately leading to catastrophic failure.

While experiments offer the most direct and reliable insights, the inherent limitation of experimental techniques to simultaneously and in situ detect internal damage necessitates the integration of finite element simulation methods to thoroughly explore the low-speed impact phenomena in composite laminates. Over the past few decades, finite element simulation has been widely used in the analysis of low-speed impacts on composite laminates. Among the various models, the Progressive Damage Model (PDM) has emerged as a leading methodology [10,11]. This model accounts for both the initiation of damage and the subsequent process of stiffness degradation. To predict damage onset, a range of damage criteria are employed. These criteria are divided into two main categories: the first category, which includes the Tsai–Wu and Tsai–Hill failure criterion [12], is limited to assessing the strength of individual laminate layers without differentiating between damage modes. Conversely, the second category of failure criteria, which encompasses the Hashin [13] and modified Hashin criteria [14], is capable of distinguishing between the four primary failure modes of laminated panels and can pinpoint damage in both the fibers and matrix of a single laminate layer.

Although first-type criteria demonstrate some accuracy in predicting the initial failure of composites, they have limitations in analyzing complex damage modes. For example, the Tsai–Wu criterion is suitable for multi-axial stress states but cannot differentiate between fiber fracture and matrix cracking. In contrast, the Hashin criterion overcomes these limitations by distinguishing between fiber and matrix failure modes, making it widely used in the damage analysis of composites under low-velocity impacts. After the initiation of damage, the simulation requires corresponding damage evolution models to depict the damage accumulation process, thereby diminishing the material’s stiffness. Several methods are available to simulate the decline in composite laminate performance during the damage evolution, such as the abrupt reduction in layer stiffness to zero, or its gradual reduction through linear or exponential reduction [15,16,17]. Zhou et al. [18] demonstrated the effectiveness of a progressive damage finite element model in examining the dynamic mechanical properties and damage evolution of composite laminates subjected to single and multiple low-speed impacts, accurately forecasting the occurrence and evolution of intralayer damage. Zhang et al. [19] utilized a three-dimensional progressive damage finite element model to simulate the low-speed impact process on laminated panels, taking into account the initiation, evolution, propagation, and interaction of various damage types. This model has proven effective in delineating the damage and failure processes of composite laminates.

Among the various typical damage modes of laminated panels, interlaminar delamination stands out as one of the most significant types of failure. To predict this delamination, scholars primarily employ two techniques: the Virtual Crack Closure Technique (VCCT) [20] and the Cohesive Zone Model (CZM) [21]. VCCT evaluates damage by simulating crack propagation paths but has limitations in predicting crack initiation and typically requires adaptive meshing techniques [22]. In contrast, CZM, based on strength and fracture energy criteria, can more comprehensively describe damage initiation and evolution, making it widely used in simulating delamination [23]. Although these methods have been implemented in many FEM software packages and support various failure criteria, including the Hashin criterion, recent studies indicate that these classical methods still have room for improvement when dealing with more complex composite damage behaviors. For example, Feng et al. [24] developed a hybrid model combining extended FEM (XFEM) and CZM to better capture the evolution of complex damage. Xu et al. [25] further enhanced damage prediction accuracy and efficiency by introducing machine learning techniques. These studies provide new perspectives and methods for damage analysis of composite laminates under low-velocity impact.

Currently, there is limited numerical simulation research on thermoplastic composite laminates, with most studies focusing on thermosetting composite laminates. However, the high cross-linking density of thermosetting resins often leads to brittle failure during impact, which restricts their broader application in engineering. In response to the urgent goals of energy conservation, pollution control, and recycling, thermoplastic matrix materials, characterized by low cost, weather resistance, recyclability, and environmental friendliness, deserve greater attention [26]. The innovative thermoplastic resin Elium^®^, developed by Arkema in Colombes, France, cures at room temperature and is easily recyclable. Compared to traditional thermosetting materials, it significantly enhances the damage tolerance of fiber-reinforced laminates, offering superior ductility and fracture toughness [27]. Therefore, this innovative material has been chosen as the matrix phase for finite element simulation.

Given the specific behavior of thermoplastic composite laminates, a comprehensive numerical modeling program is still required. This study developed a numerical simulation model for thermoplastic carbon fiber laminates under low-speed impact loads and, combined with experiments, investigated the low-speed impact performance of thermoplastic carbon fiber-reinforced composite laminates using Elium^®^ resin at different energy levels. We developed a progressive damage finite element model to simulate the behavior of these laminates under low-speed impact loads. The model uses the three-dimensional Hashin criterion to predict intra-laminate damage and incorporates a stiffness reduction matrix based on equivalent displacement to characterize the damage evolution process, with the damage constitutive model integrated into ABAQUS via the VUMAT subroutine. To predict the onset of interlaminar delamination, zero-thickness cohesive elements and a quadratic stress failure criterion were used, with the B-K criterion applied to describe the evolution of delamination damage.

To effectively assess damage to laminated composites, this study employs XCT with a spatial resolution of 41.15 μm. This high-resolution imaging technology enables precise capture of minute damage features in the laminate post impact, providing a clear view of internal damage. It aids in the detailed analysis of material behavior under low-velocity impacts and validates the accuracy of simulation results. The simulation results include force–time curves, force–displacement curves, energy absorption–time curves, three-dimensional internal damage distribution, and damage evolution processes, which were compared with impact test results and XCT scan results. The numerical simulation results are highly consistent with experimental results, validating the model’s effectiveness. Through in-depth analysis of mechanical response and damage morphology, we provide a detailed discussion of the mechanical response, damage distribution, and damage evolution process of thermoplastic carbon fiber-reinforced composite laminates under low-speed impacts.

## 2. Experimental Procedure

This study delves into composite laminates featuring the innovative thermoplastic resin Elium^®^188 as the matrix, and carbon fiber woven fabric as the reinforcement phase. The specimens were fabricated utilizing the Vacuum-Assisted Resin Infusion (VARI) process. To ascertain the low-speed impact response characteristics of these specimens across various energy levels, drop-weight impact tests were conducted.

The innovative thermoplastic resin Elium^®^188 used in this study was supplied by Arkema, a company based in France. The carbon fiber woven fabric was purchased from a company called Easy Composites located in China.

### 2.1. Materials

The raw materials employed in the fabrication of thermoplastic carbon fiber-reinforced composite laminates are specified as follows:

(1) The matrix phase is composed of Elium^®^188 thermoplastic resin, sourced from Arkema in France, combined with a benzoyl peroxide (BPO) initiator in a mass ratio of 100:2.
(2) The fiber phase is constituted by a ProFinishTM carbon fiber bidirectional woven fabric, with an areal density of 200 g/m^2^.

### 2.2. Preparation

**Figure 1:**
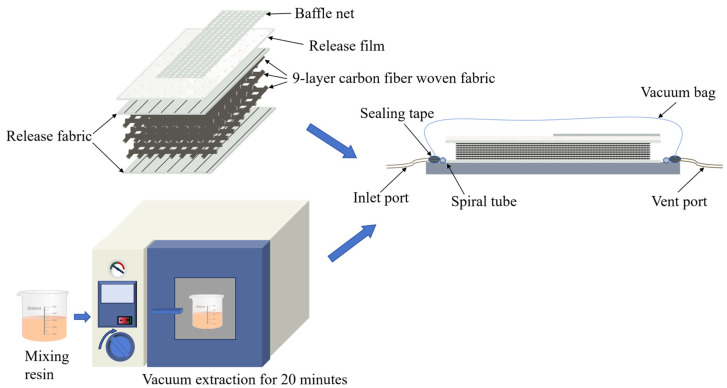
Schematic diagram of preparation process.

Laminates were fabricated using the Vacuum-Assisted Resin Infusion (VARI) process, as illustrated in Figure 1. The Elium^®^188 resin was blended with BPO in a mass ratio of 100:2, thoroughly stirred, and subsequently introduced into a vacuum chamber. The mixture was subjected to vacuum for 20 min to eliminate air bubbles from the solution. Subsequently, carbon fiber bidirectional woven fabrics were meticulously stacked in nine layers at 0 degrees. The mold cavity was arranged from top to bottom in the sequence of flow media, release film, peel ply, woven fabric, and peel ply. The mold cavity was then sealed with a vacuum bag and sealing tape, and a vacuum was applied to facilitate resin flow for 35 min. Upon completion of the resin flow, the mold was transferred to an oven for curing. The curing temperature for the thermoplastic carbon fiber-reinforced composite laminates discussed in this paper was 40 °C, with a curing duration of 3 h. The prepared pieces were precisely cut into standard test specimens measuring 100 mm × 100 mm × 2.25 mm using a water jet cutter.

### 2.3. Testing

**Figure 2:**
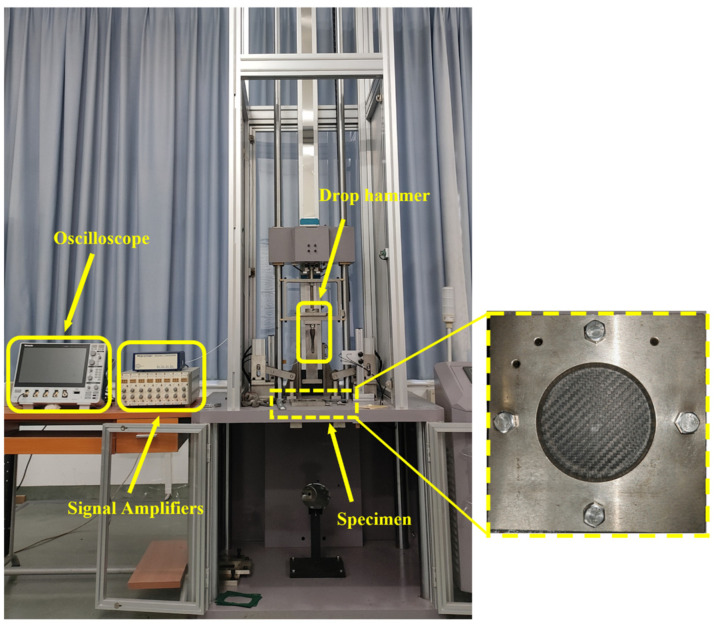
Low-velocity impact devices.

The low-velocity impact tests were conducted in strict accordance with the standard test ASTM D7136 using a drop-weight impact tester as shown in Figure 2. The 6.27 kg drop weight used in the testing featured a hemispherical hammer head at the front with a diameter of 12.7 mm. The impact tests were carefully designed at energy levels of 5 J, 10 J, 15 J, 20 J, and 25 J. Throughout the tests, the impact velocity and force versus time curves were continuously recorded to capture the dynamics of the impact process.

## 3. Numerical Model

### 3.1. Finite Element Model

A finite element model of thermoplastic carbon fiber-reinforced composite laminates subjected to low-speed impact was developed using the ABAQUS finite element modeling software. The initiation and evolution of both intralaminar and interlaminar damage were programmed via the VUMAT subroutine to conduct progressive damage analysis. This methodology facilitated the assessment of the mechanical response and damage characteristics of the laminates under low-speed impact conditions.

#### 3.1.1. Assembly

**Figure 3:**
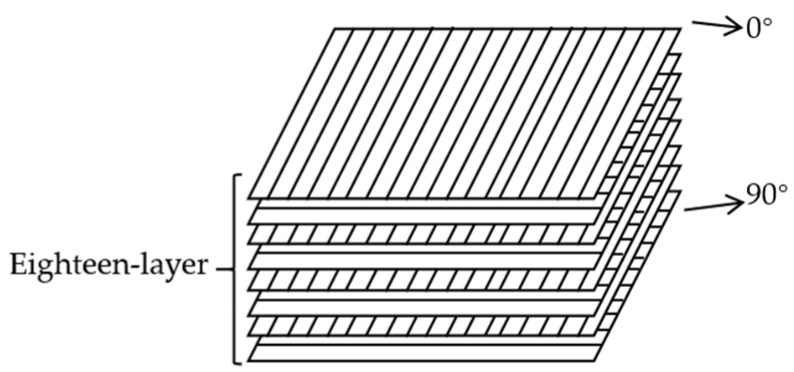
Schematic diagram of the laminate stacking sequence in the finite element model.

**Figure 4:**
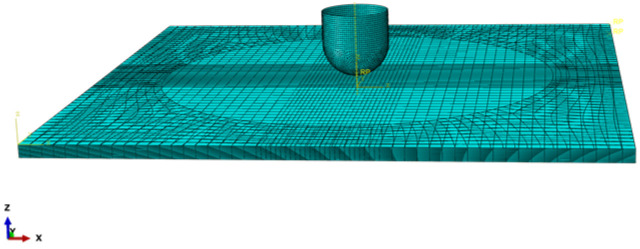
Finite element modeling of low-velocity impact.

A finite element model of the thermoplastic carbon fiber-reinforced composite laminates was meticulously established based on actual experiments, featuring a laminate stacking sequence of [0, 90]_9 s_ to replicate the nine layers of bidirectional carbon fiber woven fabric employed in the experiments, as shown in Figure 3, with dimensions of 100 mm × 100 mm × 2.25 mm. The composite laminate was placed on a fixture with a central opening diameter of 75 mm. The fixture was set with fixed constraints, while the four edges of the laminate were unconstrained in all degrees of freedom. The impactor head, a hemisphere with a diameter of 12.7 mm and a mass of 6.27 kg, was set to move in the negative y-axis direction, with the other five degrees of freedom being restricted. Figure 4 illustrates the three-dimensional finite element model of the composite laminate under low-speed impact conditions.

#### 3.1.2. Meshing

Due to the presence of stress in the thickness direction of the laminate during the actual impact process, the intralaminar elements of the laminate are modeled using C3 D8 R solid elements, with a stiffness relaxation hourglass control method selected to improve computational accuracy. Zero-thickness eight-node COH3 D8 cohesive elements are strategically inserted between layers to facilitate the analysis of delamination during the low-speed impact process. The cohesive elements share nodes with adjacent solid elements to ensure continuity of displacement.

In consideration of mesh sensitivity and with the goal of maintaining solution accuracy while enhancing computational efficiency, the composite laminate was discretized with a refined approach: the impact region was subjected to a refined mesh size of 20 mm × 20 mm (yielding an element size of 0.8 mm × 0.8 mm), whereas the remaining areas were assigned relatively coarse meshes (with element sizes of 0.8 mm × 2 mm and 2 mm × 2 mm). Due to the much higher stiffness of the hammerhead and fixture compared to the laminate thickness direction, discrete rigid bodies were employed. The low-speed impact finite element model described in this study comprises a total of 76,050 C3 D8 R elements and 33,800 COH3 D8 elements.

#### 3.1.3. Contact Settings and Material Properties

In the model, the contact pairs that necessitate consideration for contact calculations encompass the laminate surface in contact with the fixture, the laminate surface in contact with the impactor, the interior of the laminate in contact with the impactor, and the adjacent layer elements that engage upon the deletion of cohesive elements between layers due to complete delamination. The contact type employed is general contact, with hard contact specified for normal directions and a friction coefficient of 0.3 applied for tangential contact.

Table 1 presents the elastic and strength parameters of the laminate layers, as well as the interlayer interface parameters, which were derived from the literature [28,29]. Local coordinate systems were utilized to define material layup directions when configuring the properties of the intralayer elements.

### 3.2. Damage Modeling

The primary damage modes exhibited by composite laminates under low-speed impact are categorized into two types: intralaminar damage and interlaminar delamination damage. Intralaminar damage includes fiber failure and matrix failure. A progressive damage model has been developed to simulate this low-speed impact damage process, specifically incorporating damage initiation criteria and damage evolution models.

#### 3.2.1. Failure Criteria

Due to the three-dimensional stress state of the intralaminar material elements during the impact process, the three-dimensional Hashin failure criterion [14], which accounts for out-of-plane stress components, is employed to establish the initiation criteria for in-plane damage of thermoplastic carbon fiber-reinforced composite laminates:

Fiber tensile failure ($\sigma_{11}\geq 0$):

(1) $$F_{ft}={\left(\frac{\sigma_{11}}{X^{T}}\right)}^{2}+{\left(\frac{\sigma_{12}}{S_{12}}\right)}^{2}+{\left(\frac{\sigma_{13}}{S_{13}}\right)}^{2}\geq 1$$

Fiber compression failure ($\sigma_{11}<0$):

(2) $$F_{fc}={\left(\frac{\sigma_{11}}{X^{C}}\right)}^{2}\geq 1$$

Matrix tensile failure ($\sigma_{22}+\sigma_{33}\geq 0$):

(3) $$F_{mt}={\left(\frac{\sigma_{22}+\sigma_{33}}{Y^{T}}\right)}^{2}+\frac{1}{{S_{23}}^{2}}\left({\sigma_{23}}^{2}-\sigma_{22}\sigma_{33}\right)+{\left(\frac{\sigma_{12}}{S_{12}}\right)}^{2}+{\left(\frac{\sigma_{13}}{S_{13}}\right)}^{2}\geq 1$$

Matrix compression failure ($\sigma_{22}+\sigma_{33}<0$):

(4) $$F_{mc}={\left(\frac{\sigma_{22}+\sigma_{33}}{{2\;S}_{23}}\right)}^{2}+\left[{\left(\frac{Y_{c}}{{2\;S}_{23}}\right)}^{2}-1\right]\frac{\sigma_{22}+\sigma_{33}}{Y_{c}}+{\left(\frac{\sigma_{12}}{S_{12}}\right)}^{2}+{\left(\frac{\sigma_{13}}{S_{13}}\right)}^{2}\;+\frac{1}{{S_{23}}^{2}}\left({\sigma_{23}}^{2}-\sigma_{22}\sigma_{33}\right)\geq 1$$

In the formulas presented above: $X^{T}$ and $X^{C}$ represent the tensile and compressive strengths in the fiber direction of the unidirectional laminate; $Y^{T}$ and $Y^{c}$ represent the tensile and compressive strengths in the direction perpendicular to the fibers of the unidirectional laminate; $S_{12}$, $S_{13}$, and $S_{23}$ represent the shear strengths in the 1-2, 1-3, and 2-3 directions of the unidirectional laminate, respectively; and $\sigma_{ij}\;$(i, j = 1, 2, 3) represent the effective stress in each direction.

Taking into account that cohesive elements, which incorporate the traction–displacement relationship, can more accurately predict delamination damage under low-velocity impact [30,31], this type of cohesive element is introduced into the computational model. The quadratic stress failure criterion [32] is employed as the initiation criterion for delamination damage:

(5) $${\left(\frac{t_{n}}{N}\right)}^{2}+{\left(\frac{t_{s}}{S}\right)}^{2}+{\left(\frac{t_{t}}{T}\right)}^{2}=1$$

In the formula above: N represents the normal tensile strength, S and T denote the shear strengths, $t_{n}$ represents the normal tensile stress, and $t_{s}$ and $t_{t}$ represent the in-plane shear stresses.

#### 3.2.2. Damage Evolution Model

Upon meeting the damage initiation criterion, the intralayer material or interlayer interface elements do not instantaneously lose all stress and stiffness. Rather, there is a performance degradation, a decrease in stiffness coefficients, and a gradual decline in the panel’s load-bearing capacity. Therefore, it is necessary to incorporate various damage variables to define the stiffness reduction. For composite laminates, considering the intralayer material as transversely isotropic, its constitutive relationship can be articulated through a matrix encompassing five independent constants. The constitutive relationship for an undamaged unidirectional laminate, as expressed via the compliance matrix, is provided in Reference [33]:

(6) $$\varepsilon_{ij}=S\sigma_{ij}$$

(7) $$S=\left[\begin{array}{cccccc} 1/E_{11} & {-\mu }_{21}/E_{22} & {-\mu }_{31}/E_{33} & 0 & 0 & 0\\ {-\mu }_{12}/E_{11} & 1/E_{22} & {-\mu }_{32}/E_{33} & 0 & 0 & 0\\ {-\mu }_{13}/E_{11} & {-\mu }_{23}/E_{22} & 1/E_{33} & 0 & 0 & 0\\ 0 & 0 & 0 & 1/G_{12} & 0 & 0\\ 0 & 0 & 0 & 0 & 1/G_{23} & 0\\ 0 & 0 & 0 & 0 & 0 & 1/G_{31} \end{array}\right]$$

In the formulas above: $\sigma_{ij}$, $\varepsilon_{ij}$, and $E_{ij}\;$(i, j = 1, 2, 3) represent the stress, strain, and elastic modulus in each direction, respectively;

Upon the introduction of distinct damage variables for adjustment, the compliance matrix, characterized by the damage variables $\omega_{f}$ and $\omega_{m}$, can be formulated as follows:

(8) $$\left\{\begin{array}{c} S_{d11}=\frac{\Delta }{E_{11}\omega_{f}\left(1-\omega_{m}\mu_{23}\mu_{32}\right)}\\ S_{d22}=\frac{\Delta }{E_{22}\omega_{m}\left(1-\omega_{f}\mu_{13}\mu_{31}\right)}\\ S_{d33}=\frac{\Delta }{E_{33}\left(1-\omega_{f}\omega_{m}\mu_{12}\mu_{21}\right)}\\ S_{d12}=S_{d21}=\frac{\Delta }{\omega_{f}E_{11}\omega_{m}\left(\mu_{21}+\mu_{31}\mu_{23}\right)}\\ S_{d13}=S_{d31}=\frac{\Delta }{\omega_{f}E_{11}\left(\mu_{31}+\omega_{m}\mu_{21}\mu_{32}\right)}\\ S_{d23}=S_{d32}=\frac{\Delta }{\omega_{m}E_{22}\left(\mu_{32}+\omega_{f}\mu_{12}\mu_{31}\right)}\\ S_{d44}=\frac{1}{\omega_{f}\omega_{m}G_{12}}\\ S_{d55}=\frac{1}{\omega_{f}\omega_{m}G_{23}}\\ S_{d66}=\frac{1}{\omega_{f}\omega_{m}G_{31}} \end{array}\right.$$

(9) $$\left\{\begin{array}{c} \omega_{f}=1-\left(1-\omega_{ft}\right)\left(1-\omega_{fc}\right)\\ \omega_{m}=1-\left(1-\omega_{mt}\right)\left(1-\omega_{mc}\right) \end{array}\right.$$

(10) $$\Delta =1-\omega_{f}\omega_{m}\mu_{12}\mu_{21}-\omega_{m}\mu_{23}\mu_{32}-\omega_{f}\mu_{13}\mu_{31}-2\omega_{m}\omega_{f}\mu_{21}\mu_{32}\mu_{13}$$

In the formulas above: $\omega_{ft}$ and $\omega_{fc}$ represent the fiber damage variables under tensile and compressive loads, respectively; $\omega_{mt}$ and $\omega_{mc}$ represent the matrix damage variables under tensile and compressive loads.

The damage variables are expressed as:

(11) $$\omega_{I}=\frac{{\delta_{I,eq}}^{f}\left(\delta_{I,eq}-{\delta_{I,eq}}^{0}\right)}{\delta_{I,eq}\left({\delta_{I,eq}}^{f}-{\delta_{I,eq}}^{0}\right)}(\omega_{I}\in \left[0,1\right],I=ft,fc,mt,mc)$$

In Formula (12): $\delta_{I,eq}$ denotes the equivalent displacement for failure mode I, with the superscripts 0 and f indicating the initial damage matrix and the final damage matrix, respectively.

(12) $$\left\{\begin{array}{c} {\delta_{I,eq}}^{f}=\frac{2G_{I}}{{\sigma_{I,eq}}^{0}}\\ {\delta_{I,eq}}^{0}=\frac{\delta_{I,eq}}{\sqrt{F_{I}}}\\ {\sigma_{I,eq}}^{0}=\frac{\sigma_{I,eq}}{\sqrt{F_{I}}} \end{array}\right.$$

In Formula (13): $G_{I}$ represents the fracture energy density for failure mode I, $F_{I}$ represents the initial damage value for failure mode I, and $\sigma_{I,eq}$ represents the equivalent stress for failure mode I.

(13) $$G_{I}=\frac{1}{2}{\varepsilon_{I,eq}}^{f}{\sigma_{I,eq}}^{f}l_{c}$$

In Formula (14): ${\sigma_{I,eq}}^{f},{\varepsilon_{I,eq}}^{f}$ represent the equivalent peak stress and equivalent the failure strain for failure mode I, respectively; $l_{c}$ represents the characteristic length of the element.

Regarding the evolution of delamination damage, the Benzeggagh–Kenane (B-K) criterion for mixed-mode damage propagation is adopted [34,35]:

(14) $$G_{C}=G_{IC}+{\left[\left(G_{\mathrm{II}C}-G_{IC}\right)\left(\frac{G_{\mathrm{II}}+G_{\mathrm{III}}}{G_{I}+G_{\mathrm{II}}+G_{\mathrm{III}}}\right)\right]}^{\eta }$$

In Formula (15): $G_{IC}$ and $G_{\mathrm{II}C}$ represent the modeⅠand modeⅡinterlaminar fracture toughness, respectively, $G_{C}$ represents the total energy release rate at complete delamination, and η is the related coefficient in the B-K criterion, set at 1.45 [10].

## 4. Results and Discussion

### 4.1. Experimental Results

**Figure 5:**
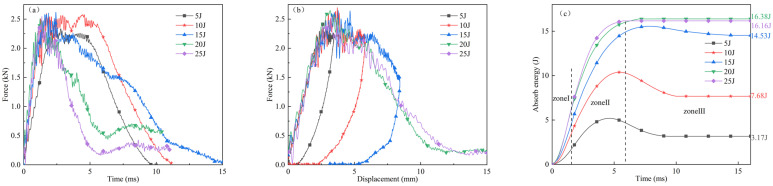
(**a**) Force–time curves; (**b**) force–displacement curves; (**c**) absorb energy–time curves of thermoplastic carbon fiber-reinforced composite laminates at different impact energies.

Figure 5 illustrates the experimental results for thermoplastic carbon fiber-reinforced composite laminates subjected to five levels of impact energy: 5 J, 10 J, 15 J, 20 J, and 25 J. Figure 5a–c depict the force–time curve, force–displacement curve, and absorbed energy–time curve, respectively.

As shown in Figure 5a, at the impact energy of 5 J, the contact force swiftly escalates upon initial contact between the hammerhead and the laminate. Subsequently, the contact force continues to rise gradually and reaches its peak. The hammerhead then begins to rebound, and the contact force gradually decreases until the hammerhead completely disengages from the laminate, resulting in a contact force reduction to zero. At the impact energy of 10 J, the contact force experiences fluctuations and ascends from zero, with the curve exhibiting violent oscillations when the contact force reaches its peak, followed by the hammerhead rebound. For impact energies of 15 J, 20 J, and 25 J, the force–time curves display pronounced sections where the peak load drops sharply, indicative of significant damage and failure within the laminate. Post drop, the contact force rebounds, attributable to the redistribution of stress within the laminate. At impact energies of 20 J and 25 J, the specimens undergo penetration. Due to the friction between the hammerhead and the hole, the contact force does not diminish to zero. Additionally, the significant friction force between the hole and the hammerhead leads to an observed increase in contact force being observed. The peak contact force of the hammerhead escalates with the increase in impact energy until it reaches the laminate’s maximum load capacity. Subsequently, even with a continued increase in impact energy, the peak contact force remains relatively stable, and the time required for the contact force to reach its peak decreases with the increase in energy.

Figure 5b presents the force–displacement curves of the laminate subjected to impact energies of 5 J, 10 J, and 15 J. Throughout the impact process, the interaction force between the hammerhead and the laminate initially generates due to their contact. As the hammerhead persists its inertial motion, the interaction force escalates gradually, and the deformation or displacement at the central contact point of the laminate also increases progressively. During this phase, the laminate experiences progressive damage, resulting in noticeable fluctuations in the curve. When the hammerhead’s velocity decelerates to zero, the unpenetrated laminate, due to its residual elastic energy, exerts this energy back onto the hammerhead, causing it to rebound. The specimens’ impacted with 5 J, 10 J, and 15 J energies all exhibited rebound behavior, whereas those impacted with 20 J and 25 J energies were penetrated and did not exhibit rebound. Throughout the impact process, as damage occurs within the laminate, the local stiffness at the impact site degrades, resulting in fluctuations in the force–displacement curve. The contact force during the impact loading phase is significantly higher than that during the rebound phase at the same displacement level. Under 10 J and 15 J impact energies, the damage process results in considerable plastic deformation, and the displacement at the end of the impact is not zero, leaving a visible dent on the surface of the laminate. Under 20 J and 25 J impact energies, as the impact energy surpasses the perforation energy threshold of the laminate, the hammerhead penetrates the laminate.

As illustrated in Figure 5c, the absorbed energy–time curve can be divided into three distinct stages: In the initial stage, the energy absorbed by the laminate is relatively low, and the slope of the curve gradually increases. In the second stage, the laminate rapidly absorbs energy through deformation, fiber and matrix cracking, as well as friction between the hammerhead and the laminate. The rate of energy absorption initially increases and subsequently decelerates, reaching a peak value. This stage corresponds to the oscillation phase of the force–time curve, wherein the kinetic energy of the hammerhead is fully absorbed. In the third stage, for laminates impacted with 5 J, 10 J, and 15 J energies, the elastic potential energy of the laminate exerts itself on the hammerhead, causing the hammerhead to rebound upward. The energy absorbed by the laminate progressively diminishes and eventually stabilizes, indicating the ultimate absorbed energy of the laminate. Under 20 J and 25 J impact energies, since the impact energy exceeds the perforation energy threshold of the laminate, the laminate is unable to absorb additional energy, and the residual impact energy is dissipated through friction and other mechanisms. When the laminate does not undergo perforation damage, the final absorbed energy of the laminate increases with the impact energy. As depicted in Figure 5c, as the impact energy increases from 5 J to 15 J, the absorbed energy value increases from 2.98 J to 14.53 J. The laminate fails to withstand the 20 J impact energy, resulting in perforation damage, indicating that the maximum impact energy the laminate can endure lies between 15 J and 20 J. The final absorbed energy of the laminate is contingent upon the laminate’s elastic and plastic deformation, the degree of fiber and matrix damage, interlaminar delamination, and friction between the hammerhead and the laminate.

**Figure 6:**
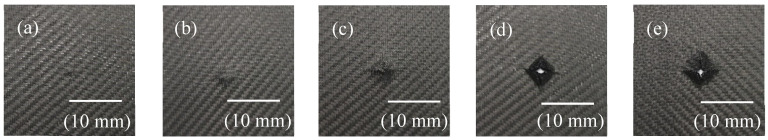
Damage morphologies on the non-impact side of thermoplastic carbon fiber-reinforced composite laminates under the impact of (**a**) 5 J; (**b**) 10 J; (**c**) 15 J; (**d**) 20 J; (**e**) 25 J.

From Figure 6, it can be seen that under a 5 J impact energy, although there is no significant damage on the non-impact side of the laminate, transverse and shear cracks have appeared, primarily indicating matrix failure. As the impact energy increases to 10 J and 15 J, these transverse and shear cracks continue to develop, and visible damage appears on the non-impact side, accompanied by noticeable delamination and fiber breakage. At 20 J and 25 J impact energies, fiber breakage further deteriorates, with the bottom of the laminate displaying a cross-shaped blooming damage pattern, along with significant delamination.

**Figure 7:**
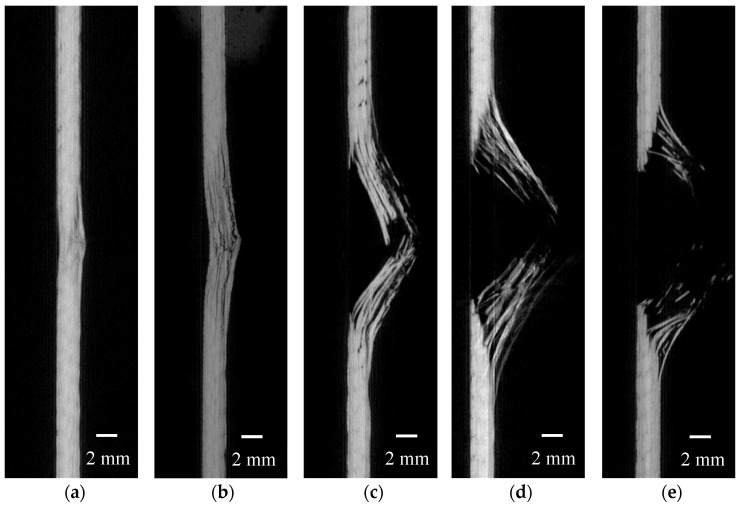
XCT scan result (**a**) 5 J; (**b**) 10 J; (**c**) 15 J; (**d**) 20 J; (**e**) 25 J.

Figure 7a–e depict the XCT scan results of the laminated composite subjected to impact energies of 5 J, 10 J, 15 J, 20 J, and 25 J, respectively, offering a lucid portrayal of the internal damage patterns within the composite material across varying impact energies. At 5 J, although the material surface only shows slight indentation and matrix failure, initial delamination has begun to appear internally. As the impact energy increases to 10 J, the depth of the indentation increases, and delamination becomes more pronounced, accompanied by slight fiber breakage. This fiber breakage indicates that the material is starting to exhibit further signs of failure under higher stress. At 15 J, delamination and cracking intensify, with significant fiber breakage and cracks extending deeper into the material, leading to a marked reduction in structural integrity. At this stage, the material’s impact resistance is nearly exhausted, with damage almost penetrating the entire thickness of the material. Under 20 J and 25 J impact energies, the damage fully penetrates the material, with extensive internal delamination, cracking, and fiber breakage, resulting in a complete loss of load-bearing capacity, indicating total failure of the material under high-energy impact. The XCT scan results clearly show that, at each impact energy level, the delamination on the non-impact side is more severe than on the impact side.

### 4.2. Finite Element Simulation Validation

**Figure 8:**
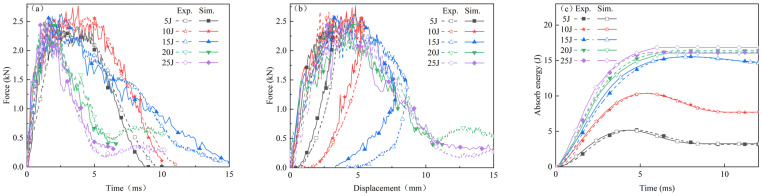
Comparison of experimental and simulated: (**a**) force–time curves; (**b**) force–displacement curves; (**c**) absorb energy–time curves.

Figure 8 juxtaposes the simulated and experimental results of force–time curves, force–displacement curves, and energy absorption–time curves for thermoplastic fiber-reinforced composite laminates subjected to impact energies of 5 J, 10 J, 15 J, 20 J, and 25 J. It is evident that the finite element model effectively simulates the mechanical response of the laminate under low-energy impact. The trends of the numerical simulation curves generally align with the experimental results. Table 2 details the peak load error analysis as predicted by the model, with an error margin confined within 5%. Table 3 provides the error analysis of the predicted absorbed energy, also maintaining an error margin within 5%.

From Figure 8b, it is evident that the maximum displacements predicted by the simulation are slightly smaller than those observed in the experimental results. This discrepancy could be attributed to the finite element model using a unidirectional laminate with a layup sequence of [0, 90]_9_ to represent the nine-ply carbon fiber bidirectional woven fabric employed in the experiment. Unidirectional fibers, characterized by their highly oriented structure, offer exceptionally high stiffness and strength along the fiber direction. In contrast, woven fiber fabrics, with fibers interwoven in multiple directions, have lower stiffness in any given direction compared to unidirectional fibers. Furthermore, in unidirectional fibers, the absence of a need to transfer load between fibers allows for more efficient load transmission along the fiber direction. In woven fiber fabrics, however, loads must be transferred at the interweaving points, which could potentially diminish load transfer efficiency and lead to increased deformation under the equivalent load. The interweaving points in woven fiber fabrics can also induce stress concentrations, potentially resulting in significant local deformation at relatively lower stress levels.

**Figure 9:**
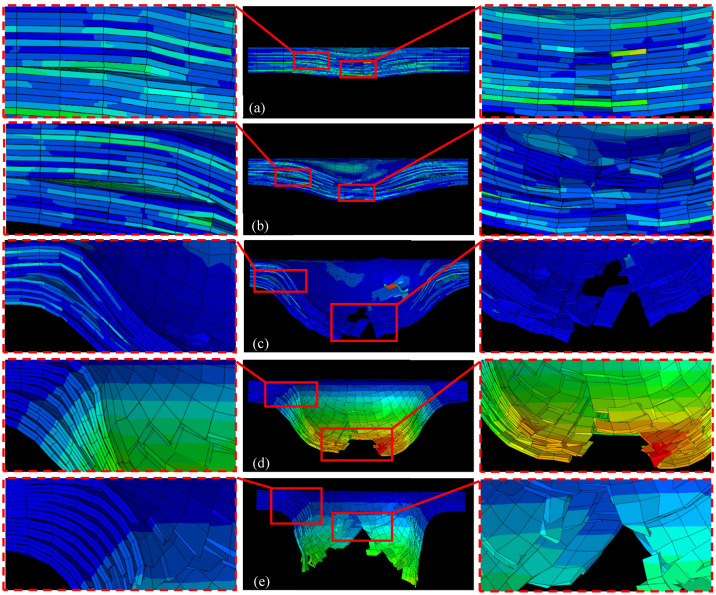
Cross-sectional damage morphologies of thermoplastic carbon fiber-reinforced composite laminates under the impact of (**a**) 5 J; (**b**) 10 J; (**c**) 15 J; (**d**) 20 J; (**e**) 25 J.

Figure 9a–e illustrate the cross-sectional damage morphology of thermoplastic carbon fiber-reinforced composite laminates subjected to impact energies of 5 J, 10 J, 15 J, 20 J, and 25 J, respectively, as derived from finite element simulations. The simulation outcomes reveal that at an impact energy of 5 J, the material exhibits slight surface indentation, with initial delamination and minor fiber breakage occurring internally. As the impact energy increases to 10 J, the indentation depth increases, delamination becomes more pronounced, and fiber breakage becomes more evident. At 15 J impact energy, delamination and cracking further expand, with cracks penetrating deeper into the material, and fiber breakage becomes more significant. Under impact energies of 20 J and 25 J, the damage extends significantly, with the internal structure almost entirely compromised, as delamination, cracking, and fiber breakage span the entire material thickness, leading to a complete loss of load-bearing capacity. A comparison with the experimental results shown in Figure 9 reveals that the simulation results closely align with the observed delamination, fiber breakage, and damage propagation characteristics.

Considering the natural variability present in experimental data, it is evident that the finite element model designed for the simulation of low-velocity impact on thermoplastic carbon fiber-reinforced composite laminates is both reasonable and effective. This model is capable of accurately predicting the mechanical response and damage characteristics of thermoplastic carbon fiber-reinforced composite laminates subjected to low-velocity impact.

### 4.3. Impact Damage Analysis

**Figure 10:**
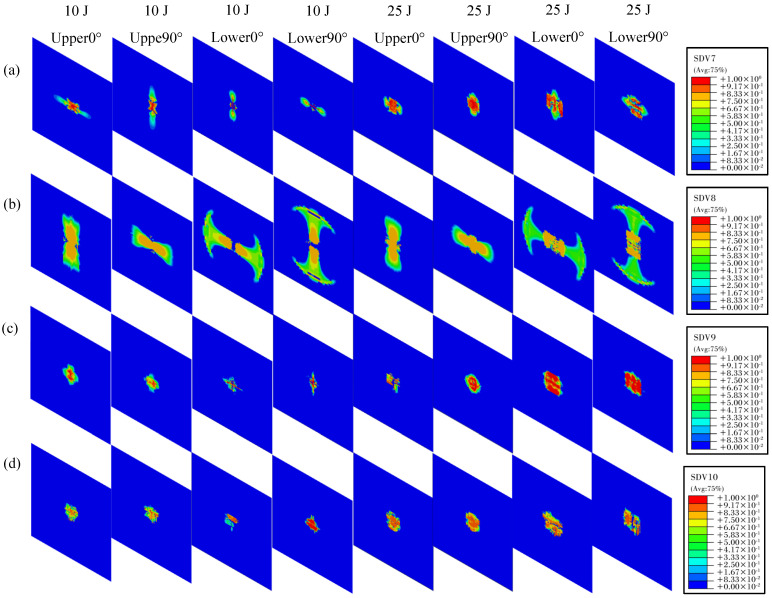
Intralaminar damage distribution on the upper and lower surfaces of thermoplastic carbon fiber−reinforced composite laminates under different impact energies: (**a**) fibre tensile; (**b**) fibre compression; (**c**) matrix tensile; (**d**) matrix compression.

**Figure 11:**
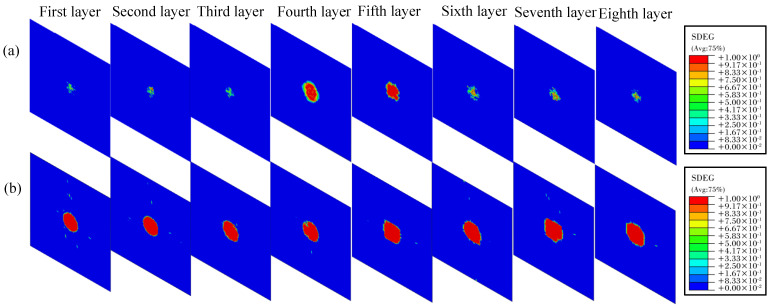
Interlaminar damage distribution of thermoplastic carbon fiber−reinforced composite laminates under different impact energies: (**a**) 10 J; (**b**) 25 J.

Figure 10 presents the damage distribution on the top and bottom surfaces of the composite laminate under impact energies of 10 J and 25 J, and Figure 11 illustrates the interlaminar delamination damage for each layer. The analysis indicates that, under varying impact energies, the laminates exhibit fiber tensile damage, matrix tensile damage, matrix compression damage, and delamination damage. The damaged elements are mainly concentrated in the central impact zone of the laminate. Due to the higher strength of the fibers compared to the matrix, the area affected by matrix damage surpasses that of fiber damage on both the top and bottom surfaces under different impact energies. As the impact energy increases, the damage area on both surfaces significantly expands. When comparing the damage zones on the top and bottom surfaces, it becomes apparent that the top surface endures more compression damage, whereas the bottom surface endures more tensile damage. This phenomenon arises from the laminate’s bending deformation when subjected to impact loads, causing the fibers and matrix on the bottom surface to bear greater tensile forces.

From Figure 11, it becomes clear that the interlaminar damage area escalates with the increase in impact energy. Generally, the damage is more severe near the neutral layer, with the bottom surface exhibiting more severe delamination than the top surface. The damage distribution across the layers is approximately symmetrical along the neutral layer. This simulation result is similar to experimental observations reported in the pertinent literature [36] and is also consistent with the XCT scan results. This trend can be explained by the combined influence of shear stress and friction: during low-velocity impact, the laminate undergoes significant bending deformation, with shear forces predominantly concentrated around the neutral layer. This results in more pronounced interlaminar damage in the fourth and fifth layers, as illustrated in Figure 11. Throughout the low-velocity impact process, stress is concentrated in the vicinity of the impact point and decreases significantly along both in-plane and through-thickness directions away from the impact point. The upper interfaces experience high pressure, generating frictional forces that mitigate delamination severity compared to the lower interfaces [34]. The relevant literature indicates that the selection of the interlaminar friction coefficient can also significantly affect the degree of delamination, an aspect that lies outside the purview of this study and will not be discussed further [36,37].

**Figure 12:**
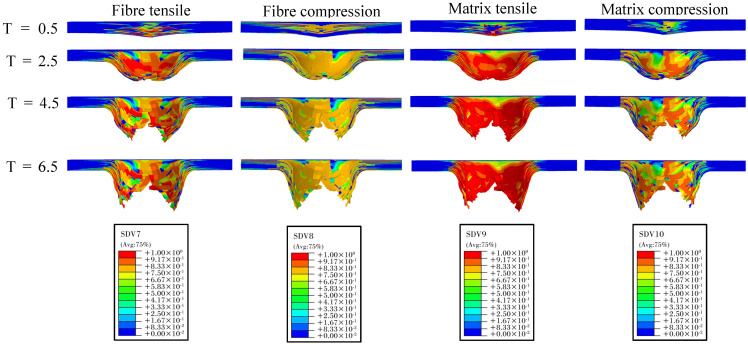
Evolution of fiber and matrix damage predicted by the model under 25 J impact energies.

Figure 12 illustrates the evolution of fiber and matrix damage under a 25 J impact energy condition. Since the impact energy exceeds the perforation threshold of the laminate, significant perforation damage occurs. At T = 0.5 ms, the matrix and fiber compression damage are primarily concentrated on the impact side, while tensile damage is mainly observed on the non-impact side away from the impact point. As time progresses to T = 2.5 ms, the tensile damage in both the matrix and fibers rapidly expands from the bottom layer towards the impact side, resulting in a significant increase in the affected area and the total damage extent. Due to the local region reaching the tensile load-bearing limit of the fibers, element deletion occurs at the bottom. At T = 4.5 ms and T = 6.5 ms, the continuous penetration of the impactor induces sequential fiber fractures, leading to severe perforation damage. The fractured fibers protrude outward with the impactor’s penetration, accompanied by significant delamination. From the comprehensive analysis of both experimental and simulation results, it is apparent that impact damage predominantly stems from delamination and intra-layer tensile failures. 

**Figure 13:**
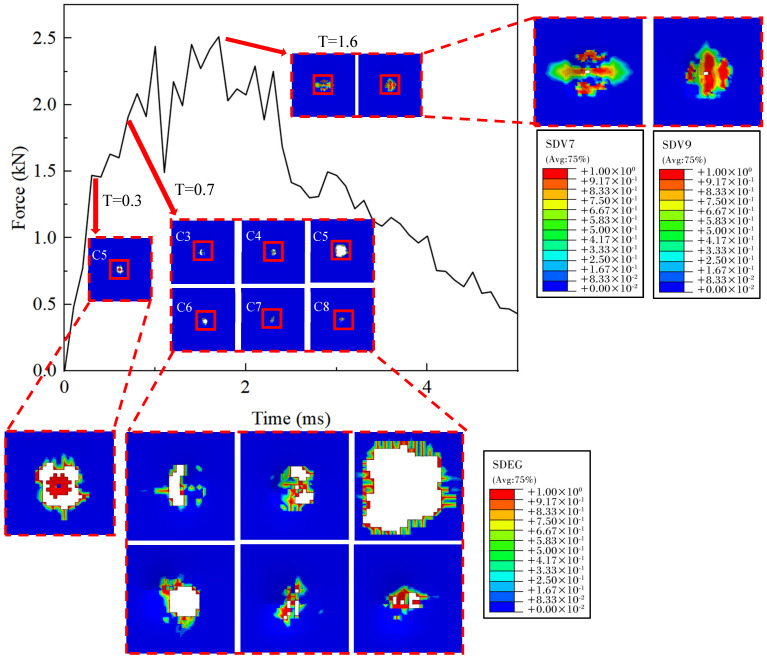
Damage evolution process of tension damage and delamination of thermoplastic carbon fiber-reinforced composite laminates under the impact of 25 J.

Figure 13 provides a detailed depiction of the evolution of intra-layer damage and delamination under a 25 J impact. At 3.0 ms, the adhesive layer of the fifth layer sustains the initial damage, leading to the removal of interlaminar cohesive elements. This event triggers the first decline in contact force and a slight reduction in the laminate’s stiffness, which is reflected in the reduced slope of the force-time curve. Between 0.4 ms and 1.0 ms, significant load drops and fluctuations can be observed in the force-time curve. During this interval, delamination propagates from the central impact center in the middle layer to both ends, eventually encompassing all interlayers. At 1.6 ms, the FRP reaches its peak load. At this juncture, the bottom fiber layer reaches its tensile load-bearing threshold, resulting in the deletion of elements and compromising the structural integrity of the laminate. Subsequently, the fibers in other layers sequentially approach their load-bearing limits, causing the overall load-bearing capacity to steadily diminish until the hammerhead penetrates through all fiber layers, culminating in the perforation of the laminate. In real-world applications, the initial damage to the laminate typically manifests as internal delamination, which may not be visibly apparent on the surface but significantly reduces the structure’s compressive strength, local stiffness, and operational lifespan. Therefore, it is imperative to ascertain the initiation and evolution of delamination at each interface. Enhancing the interlaminar bonding performance in material design can substantially improve the overall load-bearing capacity of the laminate.

## 5. Conclusions

This research has successfully formulated a progressive damage finite element model specifically designed for thermoplastic carbon fiber-reinforced composite laminates subjected to low-velocity impact loads. The model’s validity was confirmed through its congruence with experimental results. The study conducted a detailed analysis of the damage characteristics, evolution, and patterns exhibited by the laminates under varying impact energy levels. The key conclusions are succinctly summarized as follows:

(1) This study has successfully developed a progressive damage model for thermoplastic carbon fiber-reinforced composite laminates under low-velocity impact. The model adeptly simulates the mechanical response and damage characteristics of laminates constructed from woven fabrics across a spectrum of impact energies. It is capable of accurately capturing non-penetration damage behavior and can also replicate the entire process of localized damage, delamination, and failure, culminating in final penetration, under low-velocity impact conditions. The predicted peak error and absorbed energy error are maintained within a 5% margin, and the trends of the mechanical response curves closely mirror the experimental results. The damage patterns predicted by the simulation align with the findings from XCT scans, thereby further substantiating the model’s reliability.
(2) The impact damage in laminates predominantly stems from delamination and intralayer tensile failure. When the impact energy surpasses the load-bearing threshold, initial compression damage is localized on the impact side, whereas tensile damage manifests on the non-impact side. As the impact continues, tensile damage expands rapidly, and fiber fracture ensues layer by layer once the tensile limit is exceeded in the bottom layer, accompanied by extensive delamination and layer spalling.
(3) The extent of interlaminar delamination damage expands in tandem with the escalation of impact energy, with the middle layer experiencing the most extensive damage area. Delamination is notably more pronounced on the bottom surface than on the top surface, and the distribution of delamination damage across the layers is roughly symmetrical around the neutral layer. This phenomenon can be attributed to the combined effect of friction and shear stress. Initial damage typically presents as internal delamination, which may remain invisible on the surface yet substantially diminishes the structure’s compressive strength and service life. Consequently, enhancing the interlaminar bonding performance can markedly augment the overall load-bearing capacity of the laminate.

## Figures and Tables

**Table 1:** Performance parameters of thermoplastic carbon fiber plate.

Parameter	Numerical Value	Parameter	Numerical Value
$E_{11}/GPa$	57.3	$Y_{T}/MPa$	15
$E_{22}/GPa$	3	$Y_{C}/MPa$	50
$E_{33}/GPa$	3	$S_{12}=S_{13}MPa$	60
$\nu_{12}$	0.32	$S_{23}/MPa$	60
$\nu_{13}$	0.32	$K_{nn}/MPa\cdot {mm}^{-1}$	$1.37\times 10^{6}$
$\nu_{23}$	0.49	$K_{ss}=K_{tt}/MPa\cdot {mm}^{-1}$	$4.93\times 10^{5}$
$G_{12}=G_{13}/GPa$	5.1	N/MPa	62.3
$G_{23}/GPa$	4.08	S=T/MPa	92.3
$X_{T}/MPa$	650	$G_{Ic}/J\cdot m^{-1}$	0.28
$X_{C}/MPa$	240	$G_{\mathrm{II}c}=G_{\mathrm{III}c}/J\cdot m^{-1}$	0.79

**Table 2:** Peak load.

Energy	5 J	10 J	15 J	20 J	25 J
Experiment	2.39 kN	2.67 kN	2.62 kN	2.50 kN	2.54 kN
Simulation	2.41 kN	2.76 kN	2.64 kN	2.49 kN	2.51 kN
Error	0.84%	3.37%	0.76%	0.40%	1.18%

**Table 3:** Absorbed energy.

Energy	5 J	10 J	15 J	20 J	25 J
Experiment	3.17 J	7.68 J	14.53 J	16.38 J	16.16 J
Simulation	3.26 J	7.73 J	14.17 J	16.06 J	16.84 J
Error	2.84%	0.65%	2.48%	1.95%	4.21%

## Data Availability

The data presented in this study are available on request from the corresponding author.
